# Theoretical and Experimental Analysis of a New Process for Forming Flanges on Hollow Parts

**DOI:** 10.3390/ma13184088

**Published:** 2020-09-15

**Authors:** Grzegorz Winiarski, Andrzej Gontarz, Grzegorz Samołyk

**Affiliations:** Department of Computer Modelling and Metal Forming Technologies, Mechanical Engineering Faculty, Lublin University of Technology, Nadbystrzycka 38D, 20-618 Lublin, Poland; a.gontarz@pollub.pl (A.G.); g.samolyk@pollub.pl (G.S.)

**Keywords:** hollow parts, flanging, radial extrusion, aluminum alloy

## Abstract

The paper presented a new method for forming flanges on hollow parts by incremental radial extrusion. In the classic process of radial extrusion, additional rings were used to limit the free flow of material in the radial direction. The flange was formed progressively, using rings of increasing diameters. The proposed method was verified by numerical analysis and experimental tests. The numerical calculations were performed by the finite element method using the Deform-3D software package. Tubes made of aluminum alloy EN AW 6060 were used as billets. Laboratory tests were carried out using the Instron 1000 HDX testing machine. The objective of the study was to determine the validity of the proposed flange extrusion method. Results demonstrated that the new method made it possible to produce flanges with a relatively large diameter and uniform thickness, confirming the effectiveness of the proposed forming technique.

## 1. Introduction

Hollow metal flanged parts can be produced using different manufacturing techniques. These techniques can be divided into three groups: casting, machining, and forming. The selection of an appropriate method depends, among others, on the rate of production, material type, required product properties, and manufacturing costs. From the point of view of material consumption, casting and forming processes are of particularly great benefit because they allow for producing finished parts or semi-finished parts for further finishing at high material yield. Additional advantages of metal forming methods include the possibility of obtaining products with higher strength properties than those of a billet material, high efficiency, and relatively low per-unit manufacturing costs [1].

The most widely used forming methods for hollow parts are extrusion, upsetting, cross wedge rolling, rotational compression, flanging, hydroforming, and rotary swaging. Individual processes are usually dedicated to producing specified types of parts. There are also manufacturing techniques that combine several different methods. Nevertheless, the production of hollow parts is associated with many limitations that have been investigated in numerous studies, which has often led to the development of new techniques.

Aliiev et al. [2] demonstrated that the phenomenon of underfill in the direct, radial, and combined extrusion processes depended on the friction conditions and die cavity dimensions. Schiemann et al. [3] used a bar section to form a hollow flanged part; the flange was formed in several operations involving extrusion and upsetting. The authors showed that apart from local buckling of the workpiece, overlap might occur due to considerable local hardening of the material, temperature, and flow kinematics. Alves et al. [4] proposed a new method for flanging tube ends, wherein the upper and lower dies traveled at the same speed in the same direction. They also analyzed failure modes of the process, demonstrating that incompatibility between the die cavity dimensions and those of the tube could cause both local buckling of the workpiece wall and cracking of the flange formed. The study [5] presents a radial extrusion process that involves the use of an additional ring in the flange forming zone. The application of the additional tool (ring) makes it possible to form flanges with bigger diameters as well as to reduce the forming force compared to radial extrusion without the use of such a ring. In [6], the authors present radial extrusion with the use of a ring that partially limits a free flow of material in a radial direction. In effect, it is possible to form flanges with a relatively big diameter, and thickness decreasing insignificantly with increasing flange diameter. Other flanging methods presented in [7,8,9] involve extrusion with the use of a movable sleeve. In this process, the sleeve is moved in an opposite direction to that of the punch, and the tools create a closed die cavity of an increasing volume. This prevents buckling of the workpiece wall and allows for forming flanges with relatively big heights. When the process is performed in several stages or in combination with other techniques, e.g., upsetting in the tapered die cavity, it is possible to form flanges with a large diameter and height at the same time. Zhu et al. [10] proposed a process that was similar to the above-mentioned movable sleeve extrusion method. In this process, the sleeve is fixed, and the punch moves along with a counterpunch that is pressed against the face-end of the flange formed. The counterpunch is driven by a hydraulic servo-motor. Flanges can also be formed on cylindrical walls of drawn cups [11], wherein the material is deformed due to the action of shear and compressive stresses. If, however, the wall thickness and the initial distance between the punch and die are incorrect, buckling may occur. Similar failure modes occur in flange upsetting processes that are performed in either single [12] or several steps [13]. Multi-step flanging of tube ends consists of deforming a tube section in such a way to prevent buckling of the workpiece wall. Following the first stage of upsetting, the workpiece is pushed out from the lower die by a predetermined value; after that, the upsetting is continued in order to increase the flange diameter. Incremental forming methods, apart from shaping hollow products, are also used in forming cylindrical sheet metal products/tubes. These technologies can be also used in the production of products made of hard-deforming materials, such as titanium alloys [14,15].

Different techniques for producing hollow parts are rolling processes. In cross wedge rolling (CWR) [16,17,18], a part is formed using tools whose cavities are created by wedges with special geometry. The wedges cut into the material and deform it by forcing the workpiece to perform either rotary or plane motion. This process is characterized by high throughput because at least one part is produced per single working movement of the tools. On the other hand, hollow parts can be formed with tools without wedges. These processes, such as skew rolling, are used to produce tubes or bars, which are semi-finished products or finished forging [19,20].

Rotational compression is another technique that is based on workpiece rotation. In this process, the tube is put in a working space created by three rollers located concentrically on the circumference of the workpiece. The rollers rotate about their axis and translate in a radial direction at the same time, thus approaching the workpiece. Depending on the tool geometry, this process allows for forming stepped shafts [21,22,23,24,25], ball pins [26], and toothed shafts [27]. One of the numerous advantages of this process is the fact that the contact between the material deformed and the tools is short-lasting and variable. In effect, the process allows for forming parts from materials that can only be processed in narrow temperature ranges, e.g., titanium alloys [28].

Inversion is another flanging method for hollow parts. This process allows for producing parts with double walls. This technique can be used for forming both external [29] and internal [30] flanges. Flanging methods can also be classified based on the way in which the process is performed. One can distinguish here inversion with the use of a die [31] and free inversion [32]. In a free inversion, the material has no contact with the tools in the deformation zone. As a result, the friction forces on the workpiece-tool contact surface have an insignificant effect on the process, which is the main advantage of this method over inversion with the use of a die.

A completely different flanging method was proposed by Mohamed et al. [33]. In this method, the flanging of tube ends is performed on the lathe. The tube is clamped to the lathe chuck and rotated, while a ball-shaped tool is attached to the lathe carriage and fed outwards radially. Studies show that flanges with relatively big diameters can be formed on tubes with considerable wall thicknesses.

Other forming methods for producing hollow parts include hydroforming and hydroforging [34,35,36,37,38]. In these processes, a hollow blank is put between the dies, and its inner surface is subjected to the action of a high-pressure fluid, which leads to filling the die cavities. In certain cases, the impact of the high-pressure fluid can be augmented by the motion of tools, e.g., punches. Owing to its hybrid design, this process makes it possible to produce thin-walled parts with very complex geometry and high strength. An alternative to hydroforming is forging with the use of special elastic or plastic inserts. One example of such processes is the forging of hollow balls with the use of a low-melting alloy core to affect the inner wall of the workpiece [39].

Hollow parts are also widely produced by rotary swaging [40,41]. In this process, the swaging dies are located uniformly on the circumference of the workpiece and perform high-frequency movements with short strokes, thus applying compressive force onto the work piece. This forming process is predominantly used to produce axisymmetric parts that are formed by reducing the outside diameter of the billet. Hollow parts forged without the use of a mandrel are characterized by a thicker wall thickness than that of the billet. To prevent this, the forging process must be performed over a mandrel.

The literature review shows that there are numerous forming techniques for producing hollow parts. Apart from analyses of the existing techniques, studies are also conducted to extend the applicability of forming methods for hollow parts. As a result, new techniques are developed, or the existing ones are modified. This paper described a new method for forming flanges on the tube ends. This process involved incremental radial extrusion with the use of rings for limiting a free flow of material in a radial direction. The objective of the study described in this paper was to investigate the effectiveness of the proposed extrusion method. Obtained numerical and experimental results made it possible to determine the effect of process parameters on the diameter and thickness of the flange formed as well as on the stress state components that are significant in terms of potential failure modes of the process.

## 2. Materials and Methods

**Figure 1:**
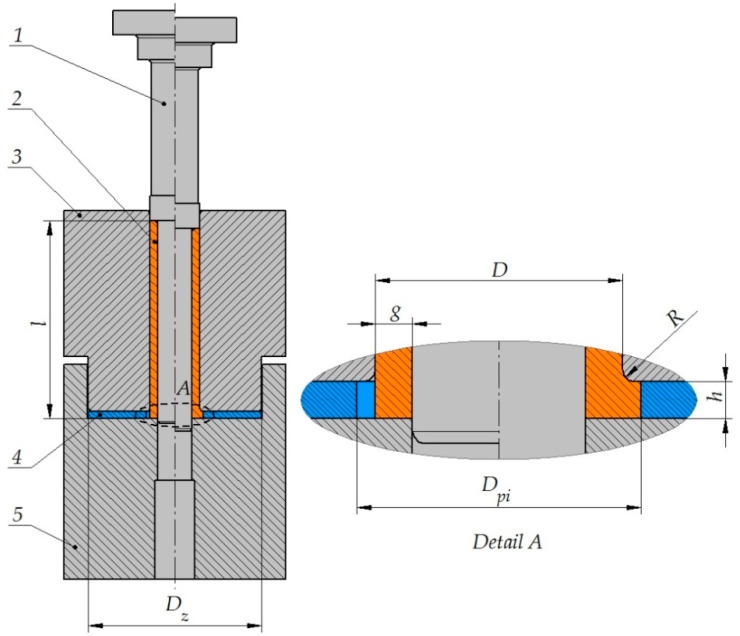
Schematic design of incremental radial extrusion of flange: 1—punch, 2—billet, 3—die, 4—ring, 5—base.

A schematic design of incremental radial extrusion of a flange is shown in Figure 1. This method is a modified version of the classical radial extrusion process, and it involves the use of additional rings. Previous studies on radial extrusion without limit rings [5,6] have shown that formed flanges have a non-uniform thickness that decreases with increasing the flange diameter. In the proposed extrusion method, a punch 1 acts on a billet 2 that is put in a die 3, which leads to filling the closed die cavity described by a height h. The die cavity is formed by a punch, die, ring 4with an inside diameter D_pi_, and a base 5. The material can flow freely in a radial direction until it comes into contact with the limit ring—until then, the flange has a variable thickness, and the die cavity is not filled completely. Since the flange diameter cannot be increased, the filling of the die cavity continues, resulting in a uniform thickness of the flange. After filling the closed die cavity with the material being deformed, the volume of the impression is increased by using the limit ring with a bigger inside diameter. After that, the extrusion process is continued as previously in order to increase the flange diameter.

**Figure 2:**
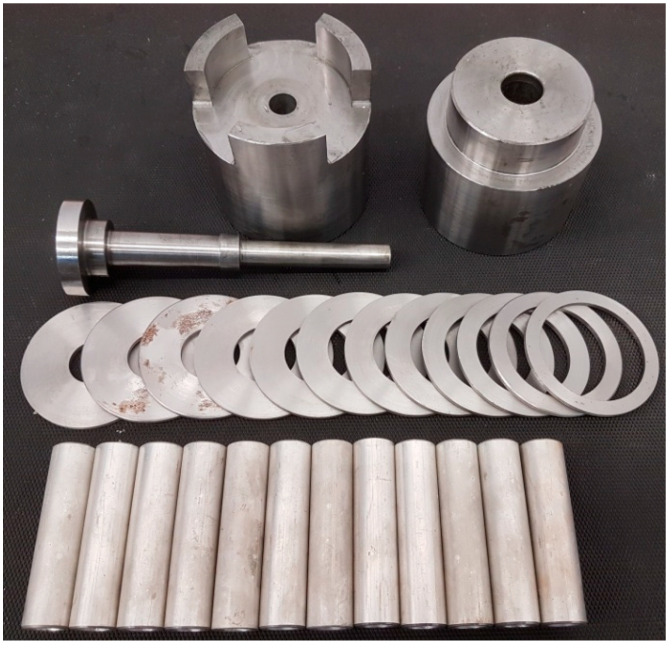
Tools and billets used in the incremental radial extrusion of the flange.

The cold incremental radial extrusion of a flange was investigated by numerical analysis and experimental tests. Simulations were performed by the finite element method using theDeform-3D software, v11. Tubes made of aluminum alloy EW AW 6060 were used, each having the dimensions D × g × l = ø20 × 3 × 80 mm. The billet was modeled as a plastic object. The 209,343 tetrahedral elements were used for the discretization of the billet. The tools were modeled as rigid objects. The tubes were annealed at 415 °C for 2 h in the LAC PK 10/12 R electric chamber furnace, produced in Rajhrad, Czech Republic [6]. The billet was heated and cooled together with the furnace. The model of the material was described by the Hollomon Equation (1). The contact between the billet and the tools was described by constant friction with the friction factor m set equal to 0.2, while the coefficient of heat transfer between the billet and the tools was set equal to 12 kW/m^2^K [6]. The heat exchange coefficient with the environment was equal to 0.02 kW/m^2^K. The initial temperature of the billet and tools was 20 °C. The punch velocity was set equal to 50 mm/min. Experiments were performed with the Instron 1000 HDX testing machine using the tools shown in Figure 2. To prevent uncontrolled movement of the die in the opposite direction to the punch movement, the die and base were clamped between two additional plates with the usage of screws.

Fundamental parameters of radial extrusion include the die cavity height, h, and the rounding radius of a die working edge, R. Previous studies [6] have shown that incorrect parameters may cause local buckling of the workpiece wall in an early stage of the extrusion process, leading to the overlapping formation. Given the above, it was assumed that the incremental radial extrusion of a flange would be performed with the use of tools having a 3-fold smaller die working edge rounding radius than the tube wall thickness, while the die cavity height would be equal to the tube wall thickness. The inside diameter D_pi_ of the rings ranged from 23 to 56 mm and was changed every 3 mm. The outside diameter D_z_ of individual rings was maintained constant at 70 mm. Numerical results were then used to determine the values of forces at which the die cavity was filled when using limit rings with different inside diameters. Experimental tests were carried out under the same conditions as those applied in the numerical analysis. Analyzed parameters are given in Table 1. The results were compared with those obtained in radial extrusion without rings [5,6].

(1) $$\sigma_{p}=147.5\times \varphi^{0.2}$$

## 3. Results and Discussion

**Figure 3:**
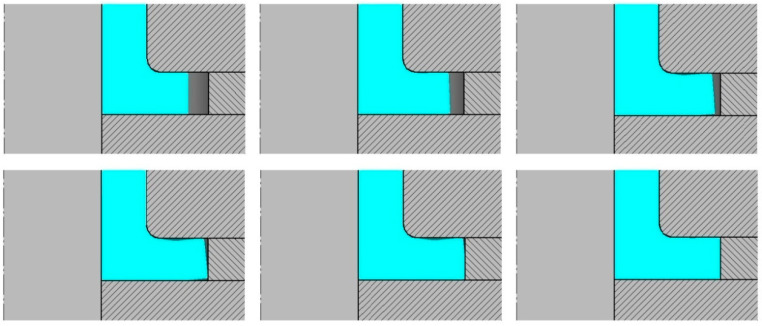
Scheme illustrating die cavity filling in the incremental radial extrusion of the flange.

**Figure 4:**
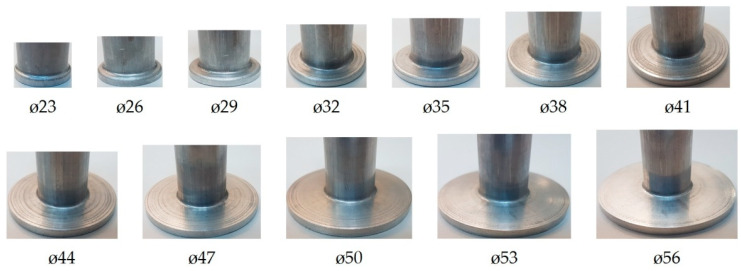
Changes in flange shape in incremental radial extrusion.

In the incremental radial extrusion of a flange, the filling of a closed die that is created, among others, by individual rings proceeds in two stages (Figure 3). In the first stage, the material flows freely in a radial direction, leading to an increase in the flange diameter; the flange has a variable thickness that is smaller than the expected one resulting from the tool dimensions. The second stage of die cavity filling begins when the workpiece comes into contact with the ring. In this stage, the flange thickness is increased to the required value. First, the lower edge of the flange comes into contact with the ring. As the extrusion process proceeds, a flange flank is formed, with the die cavity being filled in a direction from the base toward the die. At the same time, the material comes into contact with the base, but the upper edge of the flange does not contact the die until the final stage of the extrusion process. In effect, the die cavity is filled completely, which leads to the formation of a flange with a uniform thickness. Due to the fact that the process proceeds in stages, i.e., rings with increasing inside diameters are used, it is possible to obtain a flange with a higher and higher diameter (Figure 4).

**Figure 5:**
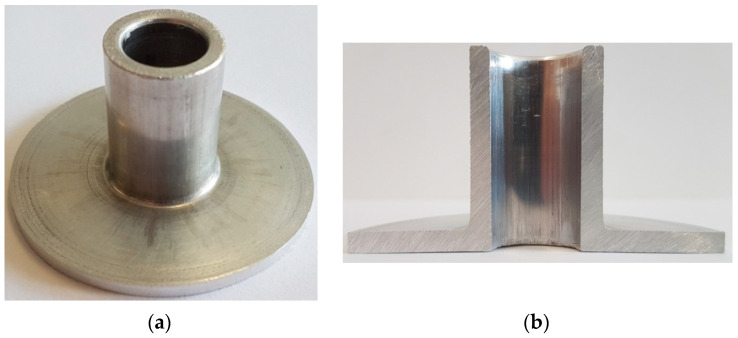
Hollow part with a 56 mm diameter flange: (**a**) perspective view, (**b**) axial section.

**Figure 6:**
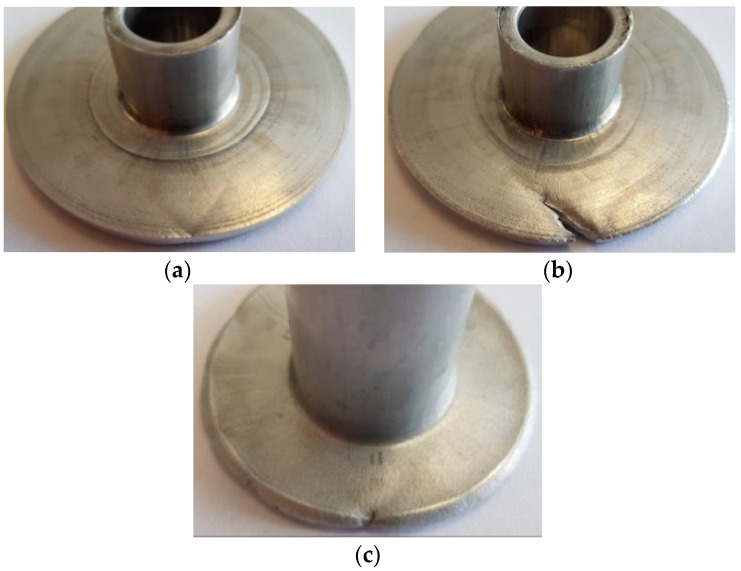
Hollow part with a flange produced by (**a**) incremental radial extrusion—crack formation, (**b**) incremental radial extrusion—visible crack, (**c**) radial extrusion without rings—visible crack.

The experimental results demonstrate that it is possible to extrude a flange of up to 56 mm in diameter. Figure 5 shows the produced flanged hollow part. The flange has a uniform and required thickness h, and it is free from cracks. Nevertheless, if the extrusion process is continued, and the flange diameter exceeds 56 mm, cracks begin to occur in the flange. The cracking initiates on the rim when the flange achieves a diameter of about 59–60 mm (Figure 6a,b).To compare, in the flange formed by the classical extrusion process (without the use of limit rings), cracks occur at a much smaller diameter of 44 mm (Figure 6c).The results demonstrate that the use of rings limiting a free flow of material in incremental radial extrusion increases the applicability of this method in terms of maximum achievable flange diameter. This is primarily due to the stress pattern in the flange. An analysis of the principal stresses reveals that the flange is subjected to triaxial compression when the die cavity created by individual rings is completely filled. This is a fundamental difference when compared to the state of stress in the classical radial extrusion without rings, in which the cyclic triaxial compression does not occur. As a result, the proposed method makes it possible to produce flanges with a uniform thickness and bigger diameter than that obtained by extrusion without the use of a limit ring.

The initiation of cracking can be predicted using one of the available fracture criteria. One of them is the Cockcroft–Latham ductile fracture criterion that is expressed by:

(2) $$\int \frac{\sigma_{max}}{\sigma_{HMH}}d\varepsilon =C$$

where

*σ_max_* is the maximum principal stress,
*σ_HMH_* is the reduced stress according to the Huber–Mises–Hencky hypothesis,
*ε* is the strain,
*C* is the material constant.

It is assumed that the higher the value of C is, the higher the probability of cracking becomes.

**Figure 7:**
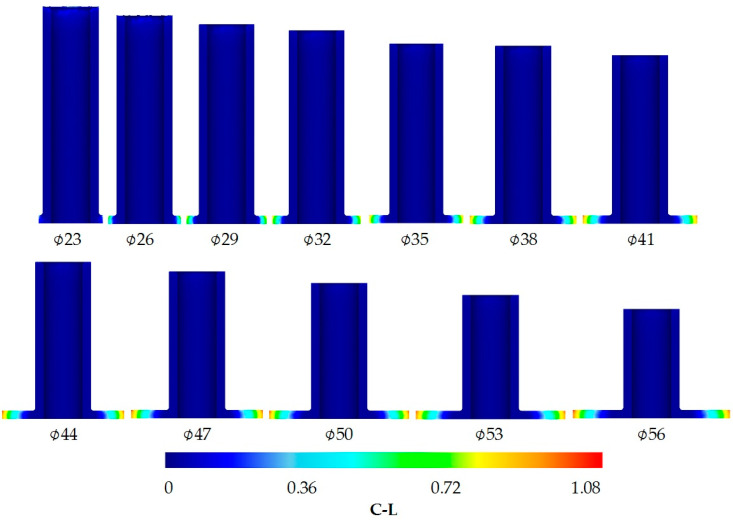
Distribution of the normalized Cockcroft–Latham ductile fracture criterion.

**Figure 8:**
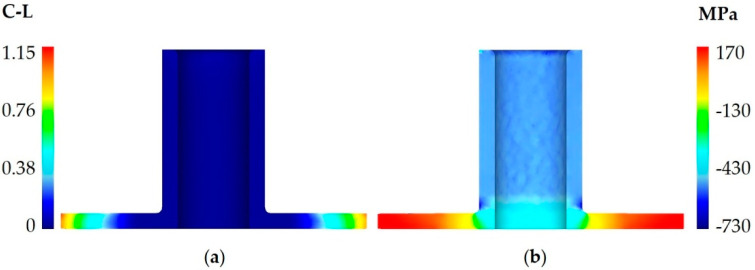
Distribution of (**a**) the normalized Cockcroft–Latham ductile fracture criterion and (**b**) circumferential stresses in a 59.5 mm diameter flange.

Figure 7 shows the distribution of the normalized Cockcroft–Latham ductile fracture criterion. It can be observed that the highest values are located on the flange rim. The value of the criterion increases with increasing the flange diameter; for a 56 mm diameter flange, it is equal to 1.08, which—as the experiments prove—is a safe value. When the material begins to lose coherence, both the maximum value of the Cockcroft–Latham criterion and the maximum circumferential stresses are located on the flange rim (Figure 8). The limit value of this criterion is about 1.15, while the circumferential stresses of about 170 MPa are tensile in nature. Therefore, the FEM analysis confirms that the first cracks occur on the flange rim. Apart from the location of cracking, its direction is equally significant, too. The circumferential stresses are relatively high for the aluminum alloy under analysis, which may indicate that cracking will be perpendicular to these stresses, i.e., it will occur in a radial direction. Nevertheless, cracking occurs in a direction that is deflected from radial by about 45° (see Figure 6b).

**Figure 9:**
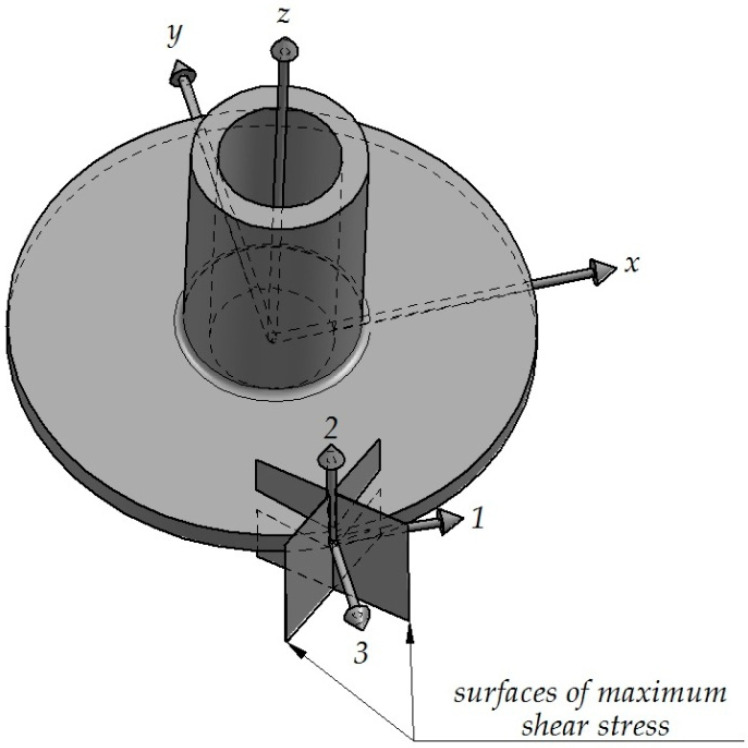
Stress pattern at the point located on the flank of a flange with a diameter at which cracking occurs.

For a detailed analysis of cracking direction, the stress state on the flange rim is investigated and described in terms of principal stresses. The investigation reveals that during the initiation of cracking, the flange is subjected to biaxial tension and compression. Apart from knowing the signs of the principal stresses, it is also important to know the orientation of the principal directions. Based on the stress components and the condition of equilibrium of internal force projections on the reference system axes {x,y,z} (Equations (3)–(5)), it is possible to determine angles that are formed by individual principal directions and the system axes {x,y,z} (Equations (6)–(8). The lettersα, β, γ, respectively, denote the angles formed by individual principal directions with the axes x,y,z of the reference system. The axes of the reference system are defined in such a way as to make the *z*-axis coincide with the symmetry axis of the flanged hollow part, while the lower surface of the flange is coplanar with the plane created by the axes x and y (see Figure 9). When the flange achieves a diameter at which cracking initiates, a random point is selected on the flank of the flange. Next, the angles formed by the principal directions and the axes of the reference system are determined for this point. Obtained results are given in Table 2 and Figure 9. The results demonstrate that the direction of the maximum principal stress σ_1_ almost coincides with that of circumferential stresses. For the analyzed case of principal stresses, the planes of the highest shear stresses (88.73 MPa) are oriented at an angle of ±45° relative to the first principal direction (Figure 9). As a result, the flange begins to crack in these planes, which is confirmed by the experimental results (Figure 6b).

(3) $$\{\begin{array}{c} \left(\sigma_{x}-\sigma_{1}\right)\times l+\tau_{xy}\times m+\tau_{xz}\times n=0\\ \tau_{yx}\times l+\left(\sigma_{y}-\sigma_{1}\right)\times m+\tau_{yz}\times n=0\\ l_{1}^{2}+m_{1}^{2}+n_{1}^{2}=1\\ l_{1}=cos\alpha_{1};\;m_{1}=cos\beta_{1};n_{1}=cos\gamma_{1} \end{array}$$

(4) $$\{\begin{array}{c} \left(\sigma_{x}-\sigma_{2}\right)\times l+\tau_{xy}\times m+\tau_{xz}\times n=0\\ \tau_{yx}\times l+\left(\sigma_{y}-\sigma_{2}\right)\times m+\tau_{yz}\times n=0\\ l_{2}^{2}+m_{2}^{2}+n_{2}^{2}=1\\ l_{2}=cos\alpha_{2};\;m_{2}=cos\beta_{2};n_{2}=cos\gamma_{2} \end{array}$$

(5) $$\{\begin{array}{c} \left(\sigma_{x}-\sigma_{3}\right)\times l+\tau_{xy}\times m+\tau_{xz}\times n=0\\ \tau_{yx}\times l+\left(\sigma_{y}-\sigma_{3}\right)\times m+\tau_{yz}\times n=0\\ l_{3}^{2}+m_{3}^{2}+n_{3}^{2}=1\\ l_{3}=cos\alpha_{3};\;m_{3}=cos\beta_{3};n_{3}=cos\gamma_{3} \end{array}$$

(6) $$\{\begin{array}{c} l_{1}=\frac{-\tau_{xy}\times m_{1}-\tau_{xz}\times n_{1}}{\left(\sigma_{x}-\sigma_{1}\right)}\\ m_{1}=n_{1}\times \frac{\tau_{yx}\times \tau_{xz}-\tau_{yz}\times \left(\sigma_{x}-\sigma_{1}\right)}{\left(\sigma_{y}-\sigma_{1}\right)\times \left(\sigma_{x}-\sigma_{1}\right)-\tau_{yx}\times \tau_{xy}}\\ n_{1}=\sqrt{\frac{1}{{\left(\frac{\frac{\tau_{xy}\times \left(\tau_{yx}\times \tau_{xz}-\tau_{yz}\times \left(\sigma_{x}-\sigma_{1}\right)\right)}{\left(\sigma_{y}-\sigma_{1}\right)\times \left(\sigma_{x}-\sigma_{1}\right)-\tau_{yx}\times \tau_{xy}}+\tau_{xz}}{\left(\sigma_{x}-\sigma_{1}\right)}\right)}^{2}+{\left(\frac{\tau_{yx}\times \tau_{xz}-\tau_{yz}\times \left(\sigma_{x}-\sigma_{1}\right)}{\left(\sigma_{y}-\sigma_{1}\right)\times \left(\sigma_{x}-\sigma_{1}\right)-\tau_{yx}\times \tau_{xy}}\right)}^{2}+1}} \end{array}$$

(7) $$\{\begin{array}{c} l_{2}=\frac{-\tau_{xy}\times m_{2}-\tau_{xz}\times n_{2}}{\left(\sigma_{x}-\sigma_{2}\right)}\\ m_{2}=n_{2}\times \frac{\tau_{yx}\times \tau_{xz}-\tau_{yz}\times \left(\sigma_{x}-\sigma_{2}\right)}{\left(\sigma_{y}-\sigma_{2}\right)\times \left(\sigma_{x}-\sigma_{2}\right)-\tau_{yx}\times \tau_{xy}}\\ n_{2}=\sqrt{\frac{1}{{\left(\frac{\frac{\tau_{xy}\times \left(\tau_{yx}\times \tau_{xz}-\tau_{yz}\times \left(\sigma_{x}-\sigma_{2}\right)\right)}{\left(\sigma_{y}-\sigma_{2}\right)\times \left(\sigma_{x}-\sigma_{2}\right)-\tau_{yx}\times \tau_{xy}}+\tau_{xz}}{\left(\sigma_{x}-\sigma_{2}\right)}\right)}^{2}+{\left(\frac{\tau_{yx}\times \tau_{xz}-\tau_{yz}\times \left(\sigma_{x}-\sigma_{2}\right)}{\left(\sigma_{y}-\sigma_{2}\right)\times \left(\sigma_{x}-\sigma_{2}\right)-\tau_{yx}\times \tau_{xy}}\right)}^{2}+1}} \end{array}$$

(8) $$\{\begin{array}{c} l_{3}=\frac{-\tau_{xy}\times m_{3}-\tau_{xz}\times n_{3}}{\left(\sigma_{x}-\sigma_{3}\right)}\\ m_{3}=n_{3}\times \frac{\tau_{yx}\times \tau_{xz}-\tau_{yz}\times \left(\sigma_{x}-\sigma_{3}\right)}{\left(\sigma_{y}-\sigma_{3}\right)\times \left(\sigma_{x}-\sigma_{3}\right)-\tau_{yx}\times \tau_{xy}}\\ n_{3}=\sqrt{\frac{1}{{\left(\frac{\frac{\tau_{xy}\times \left(\tau_{yx}\times \tau_{xz}-\tau_{yz}\times \left(\sigma_{x}-\sigma_{3}\right)\right)}{\left(\sigma_{y}-\sigma_{3}\right)\times \left(\sigma_{x}-\sigma_{3}\right)-\tau_{yx}\times \tau_{xy}}+\tau_{xz}}{\left(\sigma_{x}-\sigma_{3}\right)}\right)}^{2}+{\left(\frac{\tau_{yx}\times \tau_{xz}-\tau_{yz}\times \left(\sigma_{x}-\sigma_{3}\right)}{\left(\sigma_{y}-\sigma_{3}\right)\times \left(\sigma_{x}-\sigma_{3}\right)-\tau_{yx}\times \tau_{xy}}\right)}^{2}+1}} \end{array}$$

**Figure 10:**
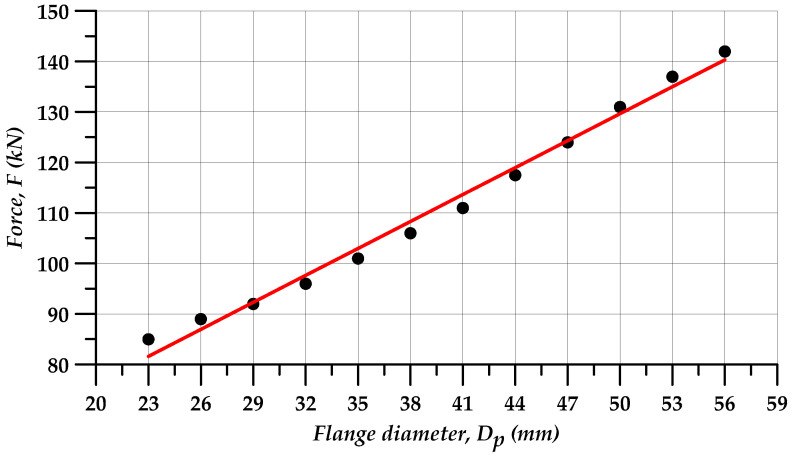
Extrusion force versus flange diameter.

The extrusion force in incremental radial extrusion rapidly increases once the material comes into contact with the ring. This is typical of closed-die forging processes in which the force increases considerably with a short length of travel of the tool. The results demonstrate that as the extrusion process advances, i.e., rings with bigger and bigger inside diameters are used, higher and higher forces are required to fill the die cavity. This can be explained by an increased volume of the material deformed and increased friction surface. Maximum extrusion forces depending on the ring diameter are given in Table 3. The forces range from 85 to 142 kN for the extrusion of 23 and 56 mm diameter flanges, respectively. The dot diagram of the analyzed force parameters in Figure 10 demonstrates that the force increases almost linearly with the increasing diameter of the extruded flange. Consequently, a pointwise approximation of the maximum forces is made, assuming that the extrusion force *F* expressed in kN will be described by a linear function, with its argument being the flange diameter *D_p_* expressed in mm (Equation (9)). The objective function described by Equation (10) is then defined, and the least-squares method is used to determine the coefficients *a* and *b*, thus yielding Equation (11) that is plotted as a red straight line in Figure 10. Given the above, it can be stated that the maximum incremental extrusion force for forming a flange with a given diameter can be roughly determined using the obtained equation.

(9) $$F\left(D_{p}\right)=a\times D_{p}+b$$

(10) $$\Omega_{F}=\sum^{\;}{\left[F_{i}-F\left(D_{p}\right)\right]}^{2}\Rightarrow minimum$$

(11) $$F\left(D_{p}\right)=1.78\times D_{p}+40.68$$

where

*D_p_* is the inside diameter of the limit ring,
*F(D_p_)* is the force as a function of limit ring diameter,
*a, b* are coefficients,
*Ω_F_* is the objective function,
*F_i_* is the value of force at i-th measurement (Table 3).

## 4. Conclusions

The results of the study lead to the following conclusions:

- the incremental radial extrusion process for hollow parts makes it possible to form flanges with their diameter bigger by 30% than that of flanges produced by extrusion without the use of rings;
- due to the favorable stress pattern, the proposed method makes it possible to produce flanges with uniform thickness, which cannot be done by the extrusion process without rings wherein the flange thickness decreases with the distance from the workpiece axis;
- due to the use of rings for limiting a free radial flow of material in flange extrusion, three-axial compressive stresses occur in the flange during filling of the closed die cavity, which is desired in terms of cracking;
- flange cracking occurs in the plane of maximum shear stresses; this plane is oriented at an angle of ±45° relative to the direction of the maximum principal stress, this direction being similar to that of circumferential stresses; in the performed research, the crack always occur in the same direction;
- the maximum forming forces increase almost linearly with increasing flange diameter.

## Figures and Tables

**Table 1:** Analyzed parameters of incremental radial extrusion of the flange (denotations according to Figure 1).

D (mm)	g (mm)	l (mm)	R (mm)	h (mm)	D_pi_ (mm)	D_z_ (mm)
20	3	80	1	3	23, 26, 29, 32, 35, 38, 41, 44, 47, 50, 53, 56	70

**Table 2:** Values of stress components at the point located on the flank of a flange with a diameter at which cracking occurs (denotations according to Figure 9).

**σ_x_** **(MPa)**	**σ_y_** **(MPa)**	**σ_z_** **(MPa)**	**τ_xy_** **(MPa)**	**τ_xz_** **(MPa)**	**τ_yz_** **(MPa)**	**σ_1_** **(MPa)**	**σ_2_** **(MPa)**	**σ_3_** **(MPa)**
171.60	−4.74	10.51	−7.71	0.25	2.74	171.93	10.98	−5.54
**α_1_** **(°)**	**β_1_** **(°)**	**γ_1_** **(°)**	**α_2_** **(°)**	**β_2_** **(°)**	**γ_2_** **(°)**	**α_3_** **(°)**	**β_3_** **(°)**	**γ_3_** **(°)**
2.49	92.49	89.95	89.62	80.29	9.71	92.47	169.97	80.28

**Table 3:** Maximum extrusion force versus inside diameter of the ring (denotations according to Figure 1).

**Measurement No.**	1	2	3	4	5	6	7	8	9	10	11	12
**D_pi_** **(mm)**	23	26	29	32	35	38	41	44	47	50	53	56
**F_i_** **(kN)**	85	89	92	96	101	106	111	117.5	124	131	137	142
